# Using Next Generation Sequencing to Study the Genetic Diversity of Candidate Live Attenuated Zika Vaccines

**DOI:** 10.3390/vaccines8020161

**Published:** 2020-04-03

**Authors:** Natalie D. Collins, Chao Shan, Bruno T.D. Nunes, Steven G. Widen, Pei-Yong Shi, Alan D.T. Barrett, Vanessa V. Sarathy

**Affiliations:** 1 Department of Microbiology and Immunology, University of Texas Medical Branch, Galveston, TX 77555, USA; natalie.d.collins.mil@mail.mil; 2 Viral Disease Branch, Walter Reed Army Institute of Research, Silver Spring, MD 20910, USA; 3 Department of Biochemistry and Molecular Biology, University of Texas Medical Branch, Galveston, TX 77555, USA; chaoshanzk@hotmail.com (C.S.); brunonunes@iec.gov.br (B.T.D.N.); sgwiden@utmb.edu (S.G.W.); peshi@utmb.edu (P.-Y.S.); 4 Wuhan Institute of Virology, Chinese Academy of Sciences, Wuhan 430071, China; 5 Department of Arbovirology and Hemorrhagic Fevers, Evandro Chagas Institute, Ministry of Health, Ananindeua, Pará 67030-000, Brazil; 6 Department of Pathology, Sealy Institute for Vaccine Sciences, Institute for Human Infections and Immunity, University of Texas Medical Branch, Galveston, TX 77555, USA; abarrett@utmb.edu

**Keywords:** next generation sequencing, *Zika virus*, genetic diversity, live-attenuated vaccine

## Abstract

*Zika virus* (ZIKV) is a mosquito-transmitted positive-sense RNA virus in the family *Flaviviridae*. Candidate live-attenuated vaccine (LAV) viruses with engineered deletions in the 3’ untranslated region (UTR) provide immunity and protection in animal models of ZIKV infection, and phenotypic studies show that LAVs retain protective abilities following in vitro passage. The present study investigated the genetic diversity of wild-type (WT) parent ZIKV and its candidate LAVs using next generation sequencing analysis of five sequential in vitro passages. The results show that genomic entropy of WT ZIKV steadily increases during in vitro passage, whereas that of LAVs also increased by passage number five but was variable throughout passaging. Additionally, clusters of single nucleotide variants (SNVs) were found to be present in the pre-membrane/membrane (prM), envelope (E), nonstructural protein NS1 (NS1), and other nonstructural protein genes, depending on the specific deletion, whereas in the parent WT ZIKV, they are more abundant in prM and NS1. Ultimately, both the parental WT and LAV derivatives increase in genetic diversity, with evidence of adaptation following passage.

## 1. Introduction

*Zika virus* (ZIKV) is a mosquito-borne flavivirus that is transmitted by *Aedes* species mosquitoes. For decades after its discovery in 1947 in Uganda, ZIKV infections were infrequent and caused mild disease, if any [1]. However, ZIKV infections spread rapidly through the Americas in 2015, leading to severe fetal neurological malformations and deficiencies, termed congenital Zika syndrome [2]. In adults, ZIKV infections cause fever, rash, and in some cases Guillain–Barré syndrome. 

Other medically important mosquito-transmitted flaviviruses are yellow fever, West Nile, Japanese encephalitis, and dengue serotype 1–4 viruses. Flavivirus genome organization consists of a single-stranded, positive-sense RNA that contains a 5’ untranslated region (UTR) followed by a single open reading frame encoding a polyprotein, and a 3’UTR, which consists of sequence components that are conserved among flaviviruses. Processing of the polyprotein results in 10 proteins: three structural proteins: capsid, pre-membrane/membrane (prM), and envelope (E), and seven nonstructural (NS) proteins: NS1, NS2A, NS2B, NS3, NS4A, NS4B, and NS5. The structural proteins make up the mature virion, whereas the nonstructural proteins function primarily in replication.

Efforts to combat ZIKV infections led to the development of several vaccine candidates, including live-attenuated vaccines (LAVs). Recombinant infectious clones of ZIKV strain FSS13025 lacking a sequence of 10–30 nucleotides in the 3’UTR have been evaluated as LAV candidates [3,4,5]. The rationale for these mutant viruses is based on recombinant live attenuated dengue viruses lacking 30–262 nucleotides in the 3’UTR. The shortest of these deletions (denΔ30) has been further developed into a candidate tetravalent LAV, which is currently undergoing phase III clinical trials (TV003/Instituto Butantan and other vaccine developers) [6,7]. The ZIKV 3’UTRΔ10 (Δ10), 3’UTRΔ20 (Δ20), and 3’UTRΔ30 (Δ30) viruses were generated as candidate LAVs in order to identify the vaccine with the best efficacy [3]. Initial in vitro and in vivo tests showed that the three ZIKV LAVs are attenuated in mice, and additional studies showed that Δ10 and Δ20 were protective in mice and nonhuman primates, as well as the fact that maternal vaccination in mice protects against ZIKV infection and transmission in utero [3,4,5,8]. In vitro passaging of LAVs is necessary in order to generate working and seed lots, and phenotypic studies have shown that 3’UTR deletion mutants retain protective abilities following in vitro passage. Sanger sequencing comparisons of the genomes after passage 0 (P0) or P5 in Vero cells indicated that Δ10, Δ20, and Δ30 retained the engineered deletions but also gained up to four amino acid substitutions in the E protein and one in the NS1 protein [3]. Importantly, no phenotypic changes were observed between P0 and P5 of Δ10. Additional testing of P4, P7, and P10 of Δ10 and Δ20 showed that up to three amino acid substitutions per clone were detected at P7 and P10; however, there was little phenotypic variability in mice between the immunogenicity of P0 and P10, suggesting that Δ10 and Δ20 are valuable LAV candidates for ZIKV [9]. In the present study, the genetic diversity of the wild-type (WT) parental virus FSS13025 and 3’UTR deletion mutants from P0 to P5 in Vero cells were examined using next generation sequencing (NGS) to establish subconsensus changes and monitor genetic diversity following passaging that may contribute to attenuation. The results demonstrated that genetic diversity increases for both WT and attenuated ZIKVs and showed clusters of genetic diversity in prM and NS1 gene regions that contribute to adaptation. Furthermore, the attenuated ZIKVs have an additional adaptation in the E gene region that may contribute to the attenuated phenotype, as it is not detected in WT parental ZIKV.

## 2. Materials and Methods

### 2.1. Viruses

WT ZIKV FSS13025 (Cambodia 2010) and 3’UTR deletion mutant-derived infectious clones [3,9,10] have been previously described, and are listed in Table 1. Briefly, ZIKV RNA was transfected into Vero cells, incubated for 9 days, harvested, and clarified by centrifugation to generate passage 0 (P0) ZIKV infectious clones. Subsequently, P0 ZIKVs were blind-passaged into fresh cultures to generate P1, then serially through P5. P1–P5 stocks were harvested from cell media at 4–5 days post-infection. 

### 2.2. Sequence Analysis

NGS determination of viral RNA genomes was performed using the Illumina platform and analyzed with Shannon entropy calculations and variant determination using Vphaser2.0, as previously detailed [11]. Briefly, RNA was extracted from culture supernatants of each virus stock, and complementary DNA libraries were constructed and sequenced using the Illumina platform. Paired-end reads were processed and de novo sequences were generated. Consensus FASTA sequences were aligned to confirm the engineered deletions. The variability (or uncertainty) at each nucleotide position was determined using Shannon entropy calculations, as previously described [11,12,13]. Single nucleotide variants (SNVs) were detected using very sensitive local conditions and controlled for false discovery and strand bias (α = 0.05).

### 2.3. Statistical Analyses

Sequence coverage and mapped read statistics were determined using Qualimap v2.1.2; non-biased SNVs were determined in Vphaser2; and means, standard error, Kruskal–Wallis with post-tests, and Spearman correlation were performed using GraphPad Prism v8. Microsoft Excel v16 was used to organize and sort the data.

## 3. Results

### 3.1. Next Generation Sequencing Results

Vero cells are widely used and accepted by regulators for the production of viral vaccines, including LAVs, and in vitro passaging of LAVs is necessary in order to generate primary and secondary seeds as well as vaccine lots. In order to study the sub-consensus level genetic diversity of the 3’UTR deletion mutants, NGS was performed and the results were compared to those acquired with the infectious clone of the WT parent virus. Every passage from P0 to P5 of WT, Δ10, Δ20, and Δ30 was sequenced using the Illumina platform. The mean coverage depth of individual viruses ranged from 540 to 10,609 (Table 1). Consensus sequence alignments confirmed that the engineered deletions were retained after passaging and that the consensus genomic WT, Δ20, and Δ30 sequences were unchanged following five passages, confirming genetic stability until P5 (Table 1). However, from P4 to P5 of Δ10, five nucleotide changes occurred (C2298U, G2306U, G2797A, C8963U, and C9293U), resulting in two amino acid substitutions (E K443N and NS1 R103K). Both substitutions were previously detected in Δ10 P5 and have been reported [3]. 

### 3.2. Shannon Entropy Increases during In Vitro Passage

**Figure 1:**
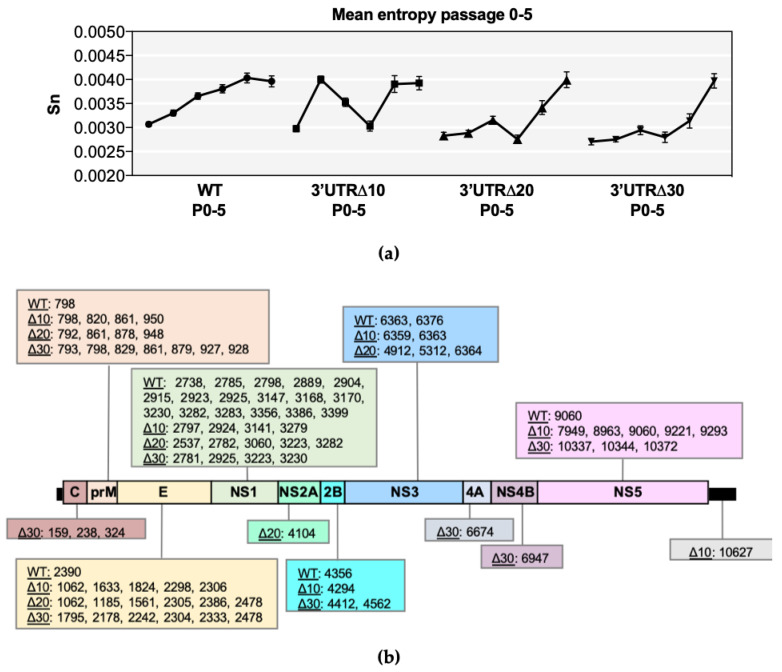
Entropy analysis of each virus sequence. (**a**) Symbols represent the mean value (+/− standard error of the mean) of positional entropy across the genome (positions 21–10750), and lines connect P0-P5 of wild-type (WT), 3’ untranslated region (UTR)Δ10, 3’UTRΔ20, and 3’UTRΔ30. (**b**) Nucleotide positions with increasing entropy values across multiple passages are shown for each *Zika virus* (ZIKV) according to the gene region/UTR. Non-parametric statistical analysis comparing the positional entropy values of each passage demonstrated that the entropy values were significant, and this is further detailed in Supplementary Table S1.

Shannon entropy was calculated in order to determine genetic diversity at each nucleotide position along each genome (Figure S1). In all ZIKVs, several high entropy positions were identified throughout the genome and collectively increased during passaging (Figure 1a). Furthermore, mean genomic entropy was higher at P4/P5 than at P0, indicating the fact that overall entropy increases during passaging (Figure 1a). Kruskal–Wallis and Dunnett’s post-tests showed significant differences across passages (*p* < 0.0001) (Table S1). The mean genomic entropy of WT gradually increased and became stabilized by P4 (Figure 1a), and high positional entropy was most abundant in the prM and NS1 genes, as previously discovered [11], but the E, NS2B, NS3, and NS5 genes had at least one position of notable entropy (Figure S1). In comparison, positional entropy values of Δ10 P0 and P1 were low; however, mean genomic entropy increased dramatically from P0 to P1, then steadily decreased through P2 and P3, followed by another sharp increase at P4 that was retained (Figure 1a). High positional entropy values clustered in prM, E, NS1, and NS5 in Δ10 P3-P5 (Figure S1). The mean genomic entropy values of Δ20 and Δ30 were similar to each other because both increased at every passage, with the exception of a slight decrease at P3 (Figure 1a). High entropy positions clustered in the C, prM, E, and NS1 genes of both Δ20 and Δ30, and were also detected in the NS3 and NS5 genes (Figure S1). 

Positions with increasing entropy values across passages were tracked as indicators of possible genetic adaptation sites and were detected in the prM, E, and NS1 gene regions of all ZIKVs (Figure 1b); however, all gene regions had at least one possible adaptation position in at least one ZIKV. Notably, WT had 17 positions in NS1 with increasing entropy, compared with only 4–5 in the NS1 of Δ10, Δ20, and Δ30. Conversely, WT ZIKV contained only one position with increasing entropy in the prM (798) and E (2390), whereas approximately 4–7 positions increased in entropy in the attenuated ZIKVs (Figure 1b). Thus, all ZIKVs may undergo adaptation in response to passaging. 

### 3.3. Variants Increase during In Vitro Passage

**Figure 2:**
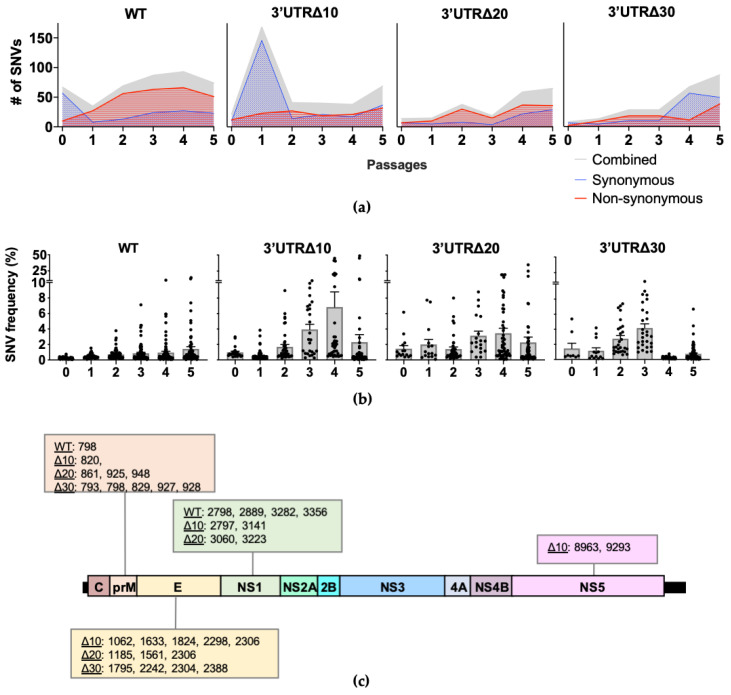
Single nucleotide variant (SNV) analysis of each virus sequence. (**a**) The total number of SNVs per passage is depicted in gray; synonymous and non-synonymous SNVs are shown in blue with dotted fill and red with horizontal line fill, respectively. Cutoff for strand bias α = 0.05. (**b**) The mean frequency (+/− standard error of the mean) of SNVs is shown across passages. Each symbol represents a SNV. (**c**) SNVs present in multiple passages with frequencies reaching at least 5% are shown according to the genome position.

Single nucleotide variants (SNVs) present in each passage of WT, Δ10, Δ20, and Δ30 were identified (Figure 2a). In WT, the number of SNVs decreased from 67 at P0 to 35 at P1, then increased gradually, peaking at 93 in P4. In WT P1-P5, the majority (approximately 74%) were non-synonymous SNVs. Interestingly, in Δ10, the number of SNVs increased from 22 at P0 to 169 at P1; P2–P4 had approximately 40 SNVs and increased to 69 at P5. Therefore, although individual positional entropy values were low in Δ10 P1, it is possible that the abundance of SNVs increased the mean genomic entropy (Figure 1a) observed for this passage. Next, both Δ20 and Δ30 had fewer SNVs than did Δ10 through P3. With the exception of Δ10 P1, the passages of attenuated ZIKVs had fewer SNVs than WT.

Across the genome, SNV frequencies increased similarly to the positional entropy values. Passaging of WT led to the emergence of SNVs in the prM, E, and NS1, as previously reported [11]; the highest frequency SNVs identified in WT were at P5: position 798 in prM at 13% and 3282 in NS1 at 15% (Figure 2b and Figure S1a). For Δ10 P1, the overwhelming majority of SNVs (162 of 169) were very low frequency (less than 1%), and by P3, SNV frequencies began to increase, including those at positions 2298, 2306, 2797, 8963, and 9293, which eventually become consensus changes in Δ10 P5 (Figure 2b and Figure S1b). SNVs in Δ20 were present at low frequency (less than 5%), except for clusters in prM, E, and NS1 (Figure 2b and Figure S1c). In Δ30, SNVs clustered in prM and E at P2 and P3 had the highest frequencies (approximately 10%), but SNV mean frequencies decreased to approximately 1% at P4 and P5 (Figure S1d). Overall, the SNV analyses show that the diversity of WT during serial passaging is dominated by non-synonymous SNVs at low frequencies, whereas the frequency of SNVs in the attenuated mutants is broader in range due to increased frequencies in the prM, E, NS1, and, to a lesser extent, NS5. 

Next, the frequencies of individual SNVs were tracked across passages to identify positions of importance for diversity and potential adaptation along the genome. SNVs increasing in frequency across multiple passages were investigated, and those reaching at least 5% frequency were present only in prM, E, NS1, and NS5 (Figure 2b and Figure S1) for a total of 5 for WT, 10 for Δ10, 9 for Δ20, and 9 for Δ30. WT had SNVs with frequencies increasing to at least 5% in prM at position 798 (13% at P5) and NS1 positions 2798 (5% at P5), 2889 (6% at P5), 3282 (15% at P5), and 3356 (7% at P5). However, when the SNV frequencies were analyzed for the attenuated mutants, the patterns showed that the highest frequency occurred at P3, P4, or P5. As expected, for Δ10, all of the nucleotide changes occurring between P4 and P5 were present as increasing SNVs in earlier passages: 2298 in E, 2306 and 2797 in NS1, and 8963 and 9293 in NS5 (Figure 2b). In Δ10, additional SNVs in prM 820; E 1062, 1633, and 1824; and NS1 3141 were detected with peak frequencies of 7%, 8%, 10%, 22%, and 45%, respectively. In Δ20, increasing frequencies peaked at P4 or P5 for SNVs in prM at positions 861, 925, and 948, (7%, 9%, and 9%, respectively); in E at positions 1185, 1561, and 2306 (17%, 35%, and 19%, respectively); and in NS1 at positions 3060 and 3223 (20% and 25%, respectively). In Δ30, all increasing high frequency SNVs peaked at P3: prM 793 (9%), 798 (11%), 829 (8%), 927 (8%), 928 (9%), and E 1795 (9%), 2242 (5%), 2304 (7%), and 2388 (6%). The SNVs increasing to at least 5% frequency across passages were only present in prM and NS1 of WT; in prM, E, NS1, and NS5 of Δ10; in prM, E, and NS1 of Δ20; and prM and E of Δ30 (Figure 2c). These results indicate that although prM and NS1 are important for the diversity of WT ZIKV, E and NS5 contribute to the diversity of attenuated mutant ZIKVs. 

### 3.4. Sequence Coverage Affects Genomic Diversity Measurements

**Figure 3:**
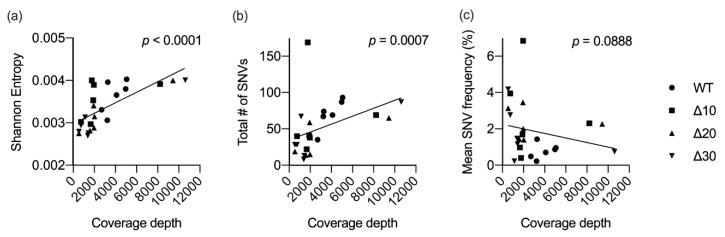
Correlation between sequence coverage and diversity. For each virus in the study (*n* = 24), mean coverage depth was plotted against mean Shannon entropy (**a**), total number of SNVs (**b**), or the mean frequency of all SNVs (**c**). Linear regression and nonparametric Spearman correlation tests were performed using GraphPad Prism V8.

As stated above, genome coverage varied among the stocks. In order to determine the effects of genomic sequence coverage on diversity, correlation tests were used. Sequence coverage was found to be significantly correlated with the mean genomic entropy (*p* < 0.0001) and with the total number of SNVs detected (*p* = 0.0007), but not with the frequency of the SNVs detected (*p* = 0.0888) (Figure 3). These data underscore the importance of providing dataset descriptions when comparing subconsensus diversity among different viruses. 

## 4. Discussion

The genetic diversity of in vitro passaged parental WT ZIKV strain FSS13025 and its candidate LAV derivatives based on 3’UTR deletion mutants, namely, Δ10, Δ20, and Δ30, were examined using NGS. Two methods of diversity analysis were applied in this study: entropy calculations and statistically significant SNV identification. Consensus sequence analysis confirmed that the three candidate LAVs did not incorporate changes in the consensus sequence following five passages in Vero cells, except that Δ10 incurs two coding substitutions after P4: E K443N (2306) and NS1 R103K (2797), as previously shown using conventional sequencing [3]. Thus, all three candidate LAVs retained the attenuating deletions following passage in a cell substrate acceptable for producing live human vaccines. Interestingly, the NS1 103 substitution was also recently detected in a live-attenuated ZIKV strain PA259249 (Panama, 2015) [14] generated through sequential passaging in HeLa cells, indicating that this position undergoes adaptation as a result of passaging in both Vero and HeLa cell culture. Less is known regarding the E 443 substitution, yet we have previously shown that American ZIKV isolates contain SNVs at this position [11]. The prM and NS1 provide genomic diversity to WT ZIKVs, and the current entropy and SNV analyses indicate that infectious clones of attenuated ZIKVs also possess high diversity in these genes and in the E gene region. 

Individual passages of each of the four ZIKVs led to multiple positions with high sequence diversity. Some positions had high entropy value or SNV frequency in a single passage, but others increased with passaging. The increase of high positional entropy and SNV frequency across passages indicated that genome optimization was occurring as a result of serial passaging, or as adaptation to Vero cells. The present study provides additional evidence for a role of prM and NS1 in adaptation of ZIKVs. Specifically for WT, nucleotide positions 798 (prM 109) and 3282 (NS1 265) were found to have SNVs that reach 13% and 15% frequency, respectively, at P5. The former SNV was previously identified in ZIKVs Dak41524 and FSS13025 [11]. Furthermore, the NS1 K265E substitution was previously characterized as a Vero adaptation that increases virus assembly but does not affect mouse virulence of ZIKVs FSS10325 and PRVABC59 [15]. In the attenuated ZIKVs, the E protein was also important for adaptation. For example, Δ10, Δ20, and Δ30 each had increasing high frequency SNVs in positions 2304 and 2306 encoding a substitution for E K443 (Figure 2c), which is conserved among related flaviviruses with the exception of Japanese encephalitis virus, which has an R instead of a K. E 443 is located in the second helix of the stem, which may play an important role in dengue virus assembly and entry [16]; however, its function in ZIKV has not been determined. Interestingly, the results of the SNV analysis varied for E 443 in the present study. In Δ10, the result was a consensus substitution of K to N at P5. In Δ20, two different SNVs (G to U at 19% and G to C at 15%) were detected at 2306, both encoding a K to N substitution that did not reach consensus level, presumably due to competition of three nucleotides (G, U, and C) at that position. In Δ30, a SNV at 2304 that encodes a K to Q substitution reached 7% frequency. In WT, SNVs at 2305 and 2306 were present at P1–P5, but the frequencies remained consistent throughout passaging at or below 1%, and were unlikely to contribute to adaptation. Additional SNVs with increasing frequencies were detected in the E gene region of Δ10, Δ20, and Δ30, but were not shared (Figure 2).

Correlation tests revealed a possible relationship between genome sequence coverage and mean entropy values and, to a lesser extent, the number of SNVs (Figure 3). The sequence coverage data for the 24 ZIKVs in this study ranged from 500 to 10,600 (Table 1). Coverage of the attenuated mutants at P5 was greater than 8000, whereas coverage of P4 was approximately 2000, yet entropy and SNV data were not proportionally increased. Therefore, coverage levels may present a limitation, but other factors, such as in vitro passage, exerted pressures on the genetic variability of passaged ZIKVs. Passaging in Vero cells may not mirror the mutational landscape that LAVs generate in the human host, yet the data are important in understanding mutation dynamics in the context of characterization of candidate Zika LAVs.

## 5. Conclusions

Overall, the genetic diversity of WT began to increase immediately during early passages and leveled after P4, indicating that WT ZIKV adapted during the passage series. Δ20 and Δ30 had mean entropy and SNV numbers that remained low through P3 then increased. Genetic diversity of Δ10 differed in that the number of synonymous SNVs and entropy increased sharply at P1 and then decreased by P2 only to rise again after P3, indicating that in vitro culture placed immediate stress on the virus sequence. Additionally, adaptation of SNVs in Δ10 led to consensus sequence changes by P5. Ultimately, all ZIKVs increased in genetic diversity after passage, but the kinetics differed between WT and attenuated ZIKVs. This study demonstrated that adaptation in mammalian cells to propagate vaccine virus includes both consensus and subconsensus changes that may contribute phenotypically, underscoring the importance of tracking genetic diversity of LAV candidates currently in development in order to determine which virus passage will be utilized for vaccine seed stocks. Ultimately, the application of NGS diversity analysis to study the genetic stability of LAVs remains under development and should be considered in the context of detailed phenotypic and immunogenicity studies.

## Figures and Tables

**Table 1:** Description and analysis of infectious clone-derived viruses used in the study.

Clone Strain	Passage	Engineered Deletion	Additional Nucleotide Changes	Coverage (+/− Standard Deviation)	Mapped Reads
**WT**	P0	none	none	3227 (+/− 1025)	701,011
**WT**	P1	none	none	2695 (+/− 970)	585,746
**WT**	P2	none	none	4105 (+/− 1244)	893,233
**WT**	P3	none	none	4967 (+/− 1426)	1,081,791
**WT**	P4	none	none	5056 (+/− 1417)	1,098,659
**WT**	P5	none	none	3276 (+/− 1112)	713,376
**3’UTRΔ10**	P0	10,650–10,659	none	1655 (+/− 514)	359,857
**3’UTRΔ10**	P1	10,650–10,659	none	1758 (+/− 433)	381,983
**3’UTRΔ10**	P2	10,650–10,659	none	1894 (+/− 510)	411,027
**3’UTRΔ10**	P3	10,650–10,659	none	748 (+/− 217)	127,882
**3’UTRΔ10**	P4	10,650–10,659	none	1959 (+/− 509)	425,289
**3’UTRΔ10**	P5	10,650–10,659	2298, 2306 ^1^, 2797 ^1^, 8963, 9293	8220 (+/− 1869)	1,787,171
**3’UTRΔ20**	P0	10,640–10,659	none	4969 (+/−1223)	1,081,689
**3’UTRΔ20**	P1	10,640–10,659	none	1968 (+/− 512)	334,354
**3’UTRΔ20**	P2	10,640–10,659	none	2019 (+/−528)	344,847
**3’UTRΔ20**	P3	10,640–10,659	none	543 (+/− 167)	92,853
**3’UTRΔ20**	P4	10,640–10,659	none	1939 (+/−594)	329,039
**3’UTRΔ20**	P5	10,640–10,659	none	9448 (+/− 2318)	2,051,441
**3’UTRΔ30**	P0	10,630–10,659	none	1385 (+/− 478)	300,206
**3’UTRΔ30**	P1	10,630–10,659	none	1433 (+/− 379)	243,275
**3’UTRΔ30**	P2	10,630–10,659	none	744 (+/− 208)	126,770
**3’UTRΔ30**	P3	10,630–10,659	none	540 (+/− 196)	95,017
**3’UTRΔ30**	P4	10,630–10,659	none	1130 (+/−350)	191,677
**3’UTRΔ30**	P5	10,630–10,659	none	10,609 (+/− 2417)	2,305,299

^1^ Changes in 2306 and 2797 led to coding substitutions E K443N and NS1 R103K, respectively.

## Data Availability

Sequence data have been deposited in the ArrayExpress database at the European Molecular Biology Laboratory- European Bioinformatics Institute under accession number E-MTAB-8905.

## Supplementary Materials

The following are available online at https://www.mdpi.com/2076-393X/8/2/161/s1: Figure S1: Entropy values and variant frequencies across the ZIKV genome. Table S1: Significance comparison of positional entropy values.
