# Supplementary material for: Using Next Generation Sequencing to Study the Genetic Diversity of Candidate Live Attenuated Zika Vaccines
Source: Vaccines (Basel). 2020 Apr 3;8(2):161. doi: 10.3390/vaccines8020161 (PMC7349499; doi:10.3390/vaccines8020161)
Supplement: Supplementary file 1 [file vaccines-08-00161-s001.pdf]

vaccines

MDPI

Supplementary Materials

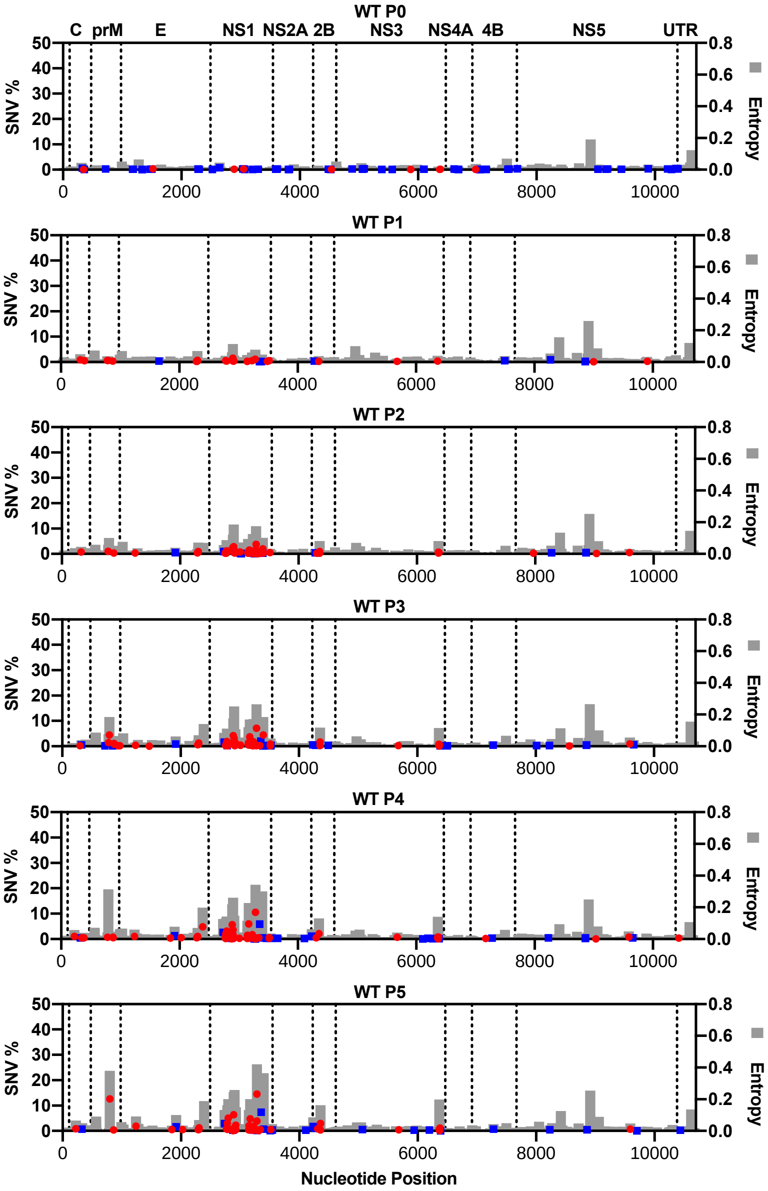

(a)

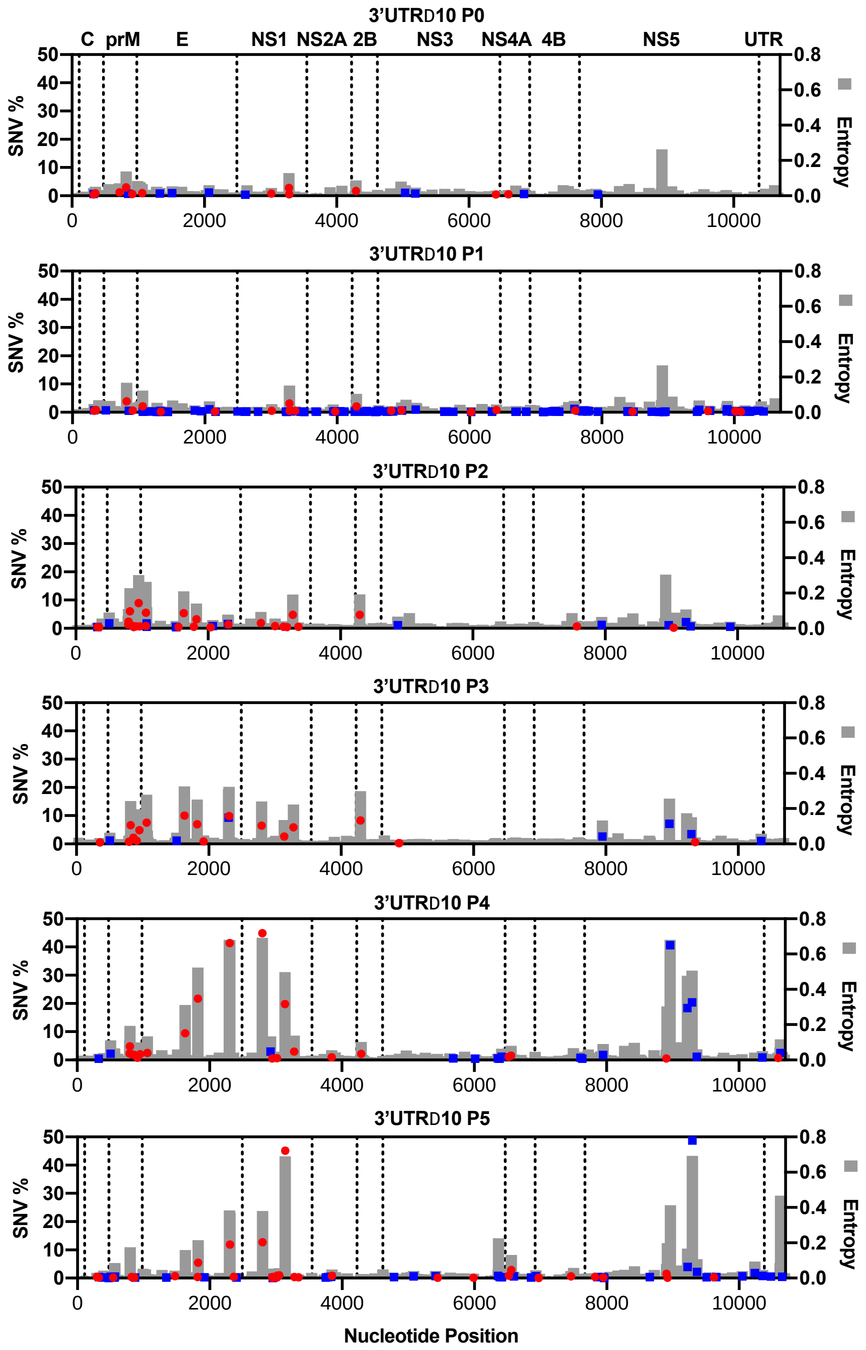

3'UTRD10 P0
C
prM
E
NS1
NS2A
2B
NS3
NS4A
4B
NS5
UTR
SNV %
Entropy
3'UTRD10 P1
3'UTRD10 P2
3'UTRD10 P3
3'UTRD10 P4
3'UTRD10 P5
Nucleotide Position

(b)

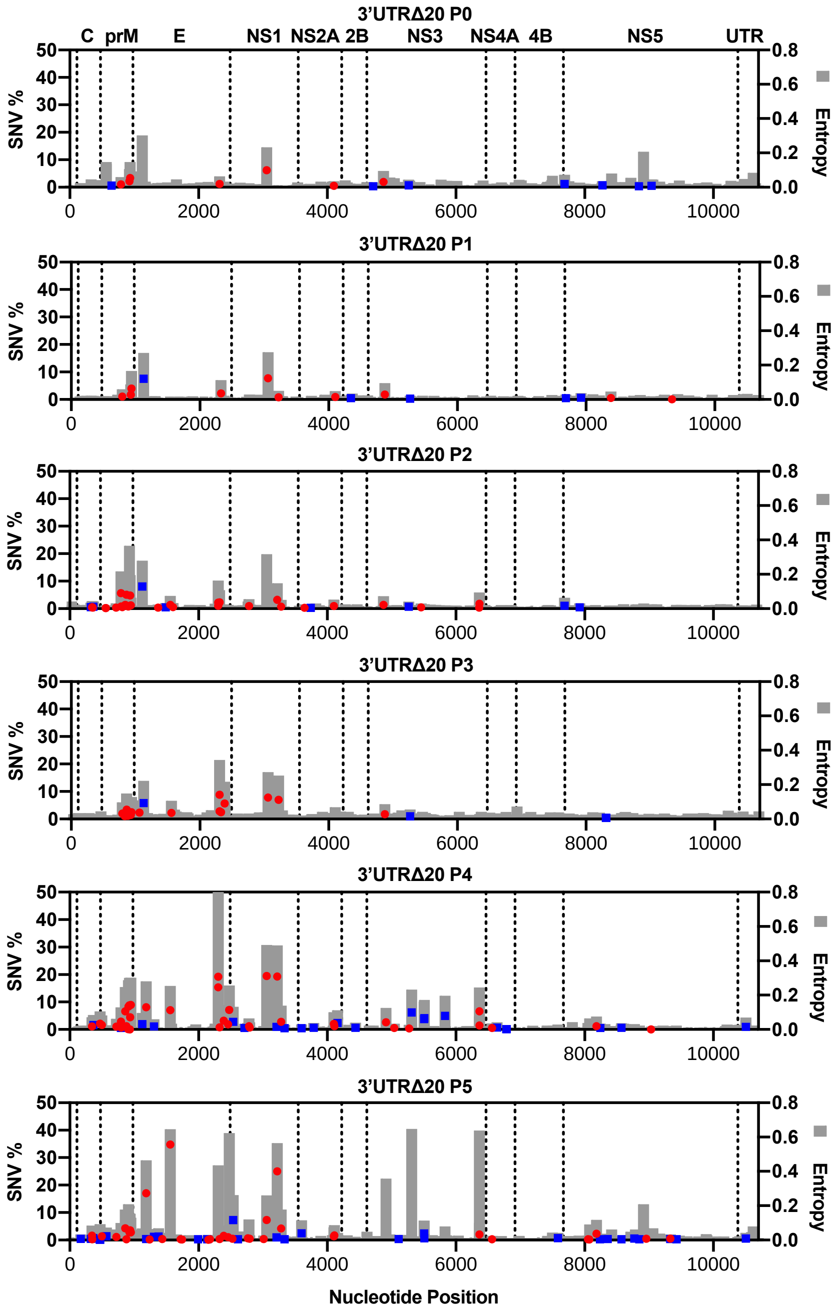
3'UTRΔ20 P0
C
prM
E
NS1
NS2A
2B
NS3
NS4A
4B
NS5
UTR
SNV %
Entropy
3'UTRΔ20 P1
3'UTRΔ20 P2
3'UTRΔ20 P3
3'UTRΔ20 P4
3'UTRΔ20 P5
Nucleotide Position

(c)

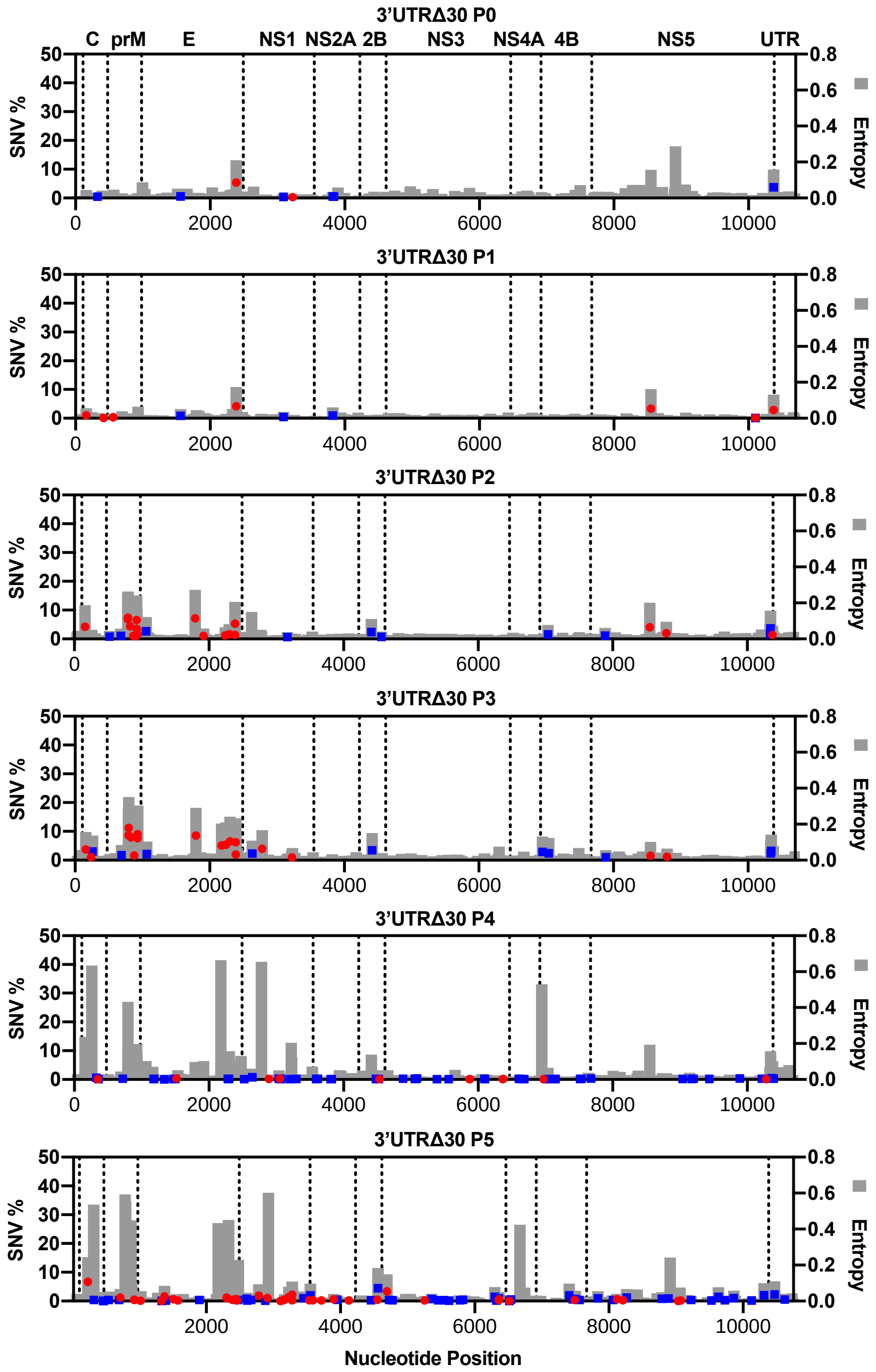

(d)

Figure 1. Entropy values and variant frequencies across the ZIKV genome. The frequency of synonymous (blue square symbols) and non-synonymous SNVs (in red circle symbols) is plotted according to nucleotide position over the entropy values (in gray bars) of each position across the genome for WT (**a**), 3′UTRΔ10 (**b**), 3′UTRΔ20 (**c**), 3′UTRΔ30 (**d**) infectious clone passages 0–5 in Vero cells. Only significant SNVs as determined by Vphaser2.0 are shown. Strand bias filter applied to SNVs: $\alpha = 0.05$.

Table 1. Significance comparison of positional entropy values. Kruskal Wallis with Dunnett's post-test p-values of mean positional entropy values.

Passages	WT	3′UTRΔ10	3′UTRΔ20	3′UTRΔ30
0 to 1	0.9375	<0.0001	<0.0001	<0.0001
0 to 2	<0.0001	<0.0001	<0.0001	<0.0001
0 to 3	<0.0001	<0.0001	<0.0001	<0.0001
0 to 4	<0.0001	<0.0001	<0.0001	0.0109
0 to 5	<0.0001	<0.0001	<0.0001	<0.0001
1 to 2	<0.0001	<0.0001	>0.9999	<0.0001
1 to 3	<0.0001	<0.0001	<0.0001	<0.0001
1 to 4	<0.0001	<0.0001	0.4621	<0.0001
1 to 5	<0.0001	<0.0001	<0.0001	<0.0001
2 to 3	0.9002	<0.0001	<0.0001	<0.0001
2 to 4	<0.0001	>0.9999	0.0023	<0.0001
2 to 5	0.0154	<0.0001	<0.0001	<0.0001
3 to 4	0.006	<0.0001	<0.0001	<0.0001
3 to 5	<0.0001	<0.0001	<0.0001	<0.0001
4 to 5	<0.0001	<0.0001	<0.0001	<0.0001
